# Association between preoperative serum sodium and postoperative 30-day mortality in adult patients with tumor craniotomy

**DOI:** 10.1186/s12883-023-03412-2

**Published:** 2023-10-04

**Authors:** Yufei Liu, Haofei Hu, Zongyang Li, Yuandi Yang, Fanfan Chen, Weiping Li, Liwei Zhang, Guodong Huang

**Affiliations:** 1 Department of Neurosurgery, Shenzhen Key Laboratory of Neurosurgery, Shenzhen Second People’s Hospital, the First Affiliated Hospital of Shenzhen University, No. 3002 Sungang west Road, Futian District, Shenzhen, Guangdong Province 518035 China; 2 Neurosurgical Department, Beijing Tiantan Hospital, Capital Medical University, No. 119, South Fourth Ring West Road, Fengtai District, Beijing, 100070 China; 3 Shenzhen University Health Science Center, Shenzhen city, Guangdong Province 518000 China; 4 Nephrological Department, Shenzhen Second People’s Hospital, The First Affiliated Hospital of Shenzhen University, Shenzhen, Guangdong Province 518035 China

**Keywords:** Sodium, Brain tumor, Craniotomy, Nonlinear, Mortality

## Abstract

**Background:**

Limited data exist regarding preoperative serum sodium (Na) and 30-day mortality in adult patients with tumor craniotomy. Therefore, this study investigates their relationship.

**Methods:**

A secondary retrospective analysis was performed using data from the ACS NSQIP database (2012–2015). The principal exposure was preoperative Na. The outcome measure was 30-day postoperative mortality. Binary logistic regression modeling was conducted to explore the link between them, and a generalized additive model and smooth curve fitting were applied to evaluate the potential association and its explicit curve shape. We also conducted sensitivity analyses and subgroup analyses.

**Results:**

A total of 17,844 patients (47.59% male) were included in our analysis. The mean preoperative Na was 138.63 ± 3.23 mmol/L. The 30-day mortality was 2.54% (455/17,844). After adjusting for covariates, we found that preoperative Na was negative associated with 30-day mortality. (OR = 0.967, 95% CI:0.941, 0.994). For patients with Na ≤ 140, each increase Na was related to a 7.1% decreased 30-day mortality (OR = 0.929, 95% CI:0.898, 0.961); for cases with Na > 140, each increased Na unit was related to a 8.8% increase 30-day mortality (OR = 1.088, 95% CI:1.019, 1.162). The sensitivity analysis and subgroup analysis indicated that the results were robust.

**Conclusions:**

This study shows a positive and nonlinear association between preoperative Na and postoperative 30-day mortality in adult patients with tumor craniotomy. Appropriate preoperative Na management and maintenance of serum Na near the inflection point (140) may reduce 30-day mortality.

## Background

Craniotomy is a basic surgical procedure for intracranial tumor resection. Unfortunately, craniotomies for intracranial tumors bring significant risks of all kinds of complications, as well as mortality [1–3]. The 30-day postoperative mortality, also known as postoperative 30-day mortality, provides an efficient and objective evaluation of the patient’s access to surgery and determining the safety of the operation and anesthesia [4, 5]. Postoperative 30-day mortality ranged from 0.95 to 8.62% based on a study of English patients with a brain tumor who underwent a craniotomy in 2008–2010 [6]. A study from a US National Surgical Quality Improvement Program (NSQIP) database reported a 30-day mortality rate of 2.6% after craniotomy for primary brain malignancies [3]. In addition, Hankinson TC et al. maintained that the 30-day postoperative mortality of diagnostic neurosurgery for a primary childhood intracranial tumor was between 1.16% and 1.72%, and the mortality of a U.S. pediatric population was in line with contemporary data from Europe [5].

According to the serum sodium (Na) levels, patients can be divided clinically into hypernatremia (Na ≥ 146 mmol/L), normal serum sodium level (Na ≥ 135 mmol/L, < 146 mmol/L) and hyponatremia (Na < 135 mmol/L) groups [7–9]. Abnormal serum Na levels have been shown to be associated with unfavorable outcomes and even increased mortality in patients admitted to the ICU after surgery [10], patients after cardiac surgery [9], patients with open abdominal surgery [11], patients with liver transplantation [12], and patients with various cancers, including pediatric brain tumors [13], head and neck squamous cell carcinoma [14], esophageal cancer [15], lung carcinoma [16], gastric malignancy [17], ovarian cancer [18], and upper tract urothelial carcinoma [19]. Plasma Na concentration disturbances expose cells to hypertonic or hypotonic stress. Although all cells of the body are affected, the clinical manifestations of hypernatremia and hyponatremia are predominantly neurologic, and rapid changes in Na concentrations in any orientation can cause permanent, severe, and occasionally lethal brain damage [20]. Hyponatremia and hypernatremia are common in some patients after neurosurgery, especially patients with sellar area lesions and thalamic lesions [21–23]. A correct diagnosis and positive management of sodium-related cerebral salt-wasting syndrome and syndrome of inappropriate secretion of antidiuretic hormone are important in the practice of dealing with neurosurgical patients [21, 24]. Accumulating studies have focused on the link between preoperative serum Na levels and postoperative prognosis [11, 14, 17, 18, 25–28].

To date, few studies have investigated the nonlinear relationship and conducted subgroup analyses between preoperative serum Na levels and 30-day postoperative mortality in cancer patients. The relationship between preoperative serum Na levels and postoperative 30-day mortality has yet to be investigated in tumor craniotomy patients older than 18 years of age. Thus, this study aimed to examine their relationship from data from the American College of Surgeons NSQIP (ACS NSQIP) database. These studies may provide clinicians with the understanding of the role of preoperative sodium in assessing short-term postoperative outcomes of tumor craniotomy patients.

## Participants and methods

### Study design

We utilized data from the ACS NSQIP database (2012–2015) for this cross-sectional study. In our study, the independent and dependent variables were serum Na before the operation and postoperative 30-day mortality, respectively.

### Data source

Jingwen Zhang et al., the authors of “Sepsis and septic shock after craniotomy: Predicting a significant patient safety and quality outcome measure [29], originally uploaded [CSV] data named [S1 Data] from the ACS NSQIP database (2012–2015). The original study was published as an open access article under the Creative Commons Attribution License, which permits unrestricted distribution, use, and reproduction. Therefore, our study could use the uploaded database for secondary analysis of nonviolation of the rights of authors.

### Participants

**Fig. 1:**
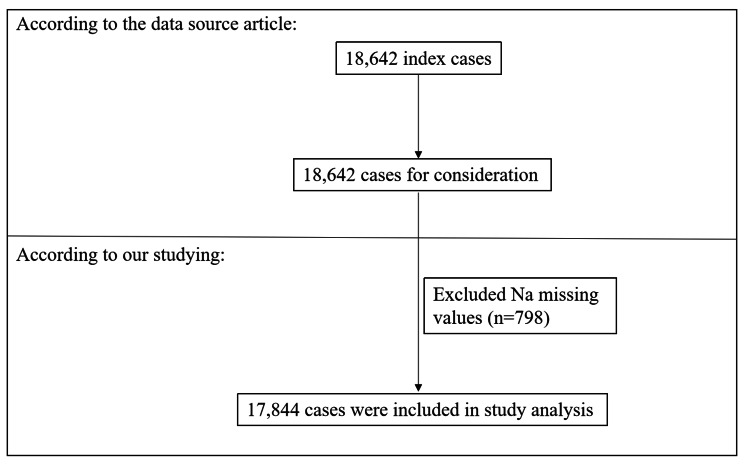
Flowchart of the study participants

The original research included 18,642 adult participants with brain tumors. A total of 17,844 participants were included in our analysis after excluding patients with missing values for preoperative serum Na (N = 798) (Fig. 1). Because our research was a secondary analysis of historical data and the original personal information was not identified, consent forms were not needed.

### Variables

Initially in the uploaded data, serum Na before surgery was recorded as a continuous variable [29]. Data collected for Na was obtained by the Current Procedural Terminology code.

### Thirty-day postoperative mortality

Originally, the authors defined 30-day postoperative mortality as mortality within the first 30 days following a surgical procedure [29]. The NSQIP tracked mortality for the first 30 postoperative days.

### Covariates

Our choice of covariates was based on previous literature reports and our clinical experience. Weight (kg) divided by height (m) squared (kg/m^2^) was used to calculate body mass index (BMI). Standard conditions were followed when collecting and treating the original data. The continuous variables in this study were as follows: indicators of preoperative blood tests (blood urea nitrogen (BUN), white blood cell (WBC) count, creatine (Cr), hematocrit (HCT), platelet (PLT) count) and BMI; and categorical variables were as follows: sex (male or female), age ranges (year-old) (18–40, 41–60, 60–80, or > 80), race (White, Asian, African American or unknown race), a history of hypertension (No, Yes), diabetes (No, Yes (noninsulin-dependent) or Yes (insulin-dependent)), smoking status (No, Yes), severe chronic obstructive pulmonary disease (COPD) (No, Yes), congestive heart failure (CHF) (No, Yes), renal failure (No, Yes), dialysis (No, Yes), steroid use for a chronic condition (No, Yes), disseminated cancer (No, Yes) and preoperative systemic sepsis (No, systemic inflammatory response syndrome (SIRS) or septic shock). The authors provided more details in the original study [29].

### Statistical analysis

The number and proportion of patients with missing values for BMI, BUN, Cr, WBC, HCT and PLT were 709 (3.97%), 798 (4.47%), 139 (0.78%), 198 (1.11%), 113 (0.63%), and 193 (1.08%), respectively. The median or mean value replaced the missing values for statistical analysis.

According to the serum Na levels, patients were divided clinically into three groups: hyponatremia (Na < 135 mmol/L), normal serum sodium level (Na ≥ 135 mmol/L, < 146 mmol/L) and hypernatremia (Na ≥ 146) groups. We reported the mean ± standard deviation (SD) (normally distributed variables) or median (interquartile range) (nonnormally distributed variables) for continuous variables; we extracted percentages and frequencies for categorical outcomes. χ^2^ tests (for categorical variables), Kruskal‒Wallis H tests or one-way ANOVAs were conducted to test for statistically significant differences among the three serum Na groups. To explore the relationship between serum Na level and postoperative 30-day mortality, distinctive univariate and multivariate binary logistic regression models were established complying with the Strengthening the Reporting of Observational Studies in Epidemiology (STROBE) statement guidelines. The models included a nonadjusted model (not adjusted for any covariates), a minimally adjusted model (only adjusted for sex and age range), and a fully adjusted model (adjusted for those variables that were significant in univariate analysis including serum WBC, BUN, Cr, PLT and HCT, sex, age range, renal failure, diabetes, severe COPD, CHF, hypertension, dialysis, disseminated cancer, preoperative systemic sepsis, steroid use) for the covariates presented in Table 1. We recorded 95% confidence intervals and effect sizes and adjusted the effect sizes when covariates were added to the model and if the OR changed by 10% or more [30].

We conducted a generalized additive model (GAM) and smooth curve fitting to address the specific link between serum Na level and postoperative 30-day mortality. If nonlinearity was detected, the inflection point was calculated using a recursive algorithm, and a piecewise binary logistic regression model with one piece on each side of the inflection point was subsequently constructed. We employed the log-likelihood ratio test to determine the most suitable model for interpreting the link between preoperative serum Na level and postoperative 30-day mortality.

We conducted subgroup analyses using a stratified binary logistic regression model for the various subgroups, including sex, a history of severe COPD, hypertension, metastatic cancer and steroid use. First, we adjusted each stratification for the factors (sex, renal failure, serum Na, BUN, Cr, WBC count, PLT count, age range, steroid use, diabetes, disseminated cancer, CHF, severe COPD, hypertension, dialysis, preoperative systemic sepsis) in addition to the stratification factor itself. Second, we tested for interactions by using the likelihood ratio test for models with and without interaction terms [31, 32].

We performed sensitivity analyses to verify the robustness of our findings. We converted serum Na level into a categorical variable based on the definitions of hyponatremia and hypernatremia. We also investigated the potential for unknown confounders of the link between serum Na level and 30-day postoperative mortality by calculating E-values [33]. All results were reported in compliance with the STROBE statement guidelines. We followed the methods of Liu et al. [34].

Statistical analyses were performed using the statistical software EmpowerStats (http://www.empowerstats.com, X&Y Solutions, Inc., Boston, MA) and the R package (http://www.R-project.org, The R Foundation). For all the tests, a two-sided p value < 0.05 was considered statistically significant.

## Results

### Study population

Demographic and clinical characteristics of the study participants are provided in Table 1. A total of 17,844 patients (47.59% male) were included in our analysis. The age (years old) distribution proportions were 15.89% (18–40), 41.54% (41–60), 39.23% (61–80) and 3.33% (> 81). The mean preoperative Na level was 138.63 ± 3.23 (mmol/L). The postoperative 30-day mortality of the included patients was 2.54% (454/17,844). Compared with those of the participants with hypernatremia, the hyponatremia group was significantly positively associated with BMI, sex, preoperative serum BUN, WBC, Cr, HCT and PLT count, race, age range, diabetes, disseminated cancer, smoking status, severe COPD, CHF, hypertension, dialysis, steroid use and preoperative systemic sepsis (all P values < 0.05), except renal failure (P values > 0.05). These results may be due to the sample size.

**Table 1:** The baseline characteristics of the participants

Na (mmol/L)	Hypernatremia group (Na ≥ 146)	Normal serum Na group (Na ≥ 135, < 146)	Hyponatremia group (Na < 135)	P value
**N**	204	15,925	1715	
**BMI (Mean ± SD)**	29.105 ± 6.701	28.813 ± 6.644	27.892 ± 6.371	< 0.001
**BUN (Median (Q1-Q3))**	17.000 (14.000-22.500)	16.000 (12.000–20.000)	18.000 (14.000–25.000)	< 0.001
**WBC (Median (Q1-Q3))**	7.700 (5.700-10.310)	8.400 (6.400–11.300)	10.900 (7.800–14.300)	< 0.001
**Cr (Median (Q1-Q3))**	0.921 ± 0.383	0.859 ± 0.419	0.890 ± 0.677	0.004
**HCT(Mean ± SD)**	37.969 ± 5.967	40.389 ± 4.718	39.653 ± 5.320	< 0.001
**PLT (Median (Q1-Q3))**	217.500 (166.000-268.250)	237.000 (195.000-284.000)	244.000 (194.000-297.000)	< 0.001
**Sex, N (%)**				< 0.001
**Male**	95 (46.569%)	7464 (46.870%)	933 (54.402%)	
**Female**	109 (53.431%)	8461 (53.130%)	782 (45.598%)	
**Age ranges (years), N (%)**				< 0.001
**18–40**	26 (12.745%)	2660 (16.703%)	150 (8.746%)	
**41–60**	79 (38.725%)	6719 (42.192%)	614 (35.802%)	
**61–80**	90 (44.118%)	6043 (37.947%)	868 (50.612%)	
**> 81**	9 (4.412%)	503 (3.159%)	83 (4.840%)	
**Race, N (%)**				< 0.001
**White**	143 (70.098%)	11,396 (71.560%)	1162 (67.755%)	
**Asian**	10 (4.902%)	467 (2.932%)	46 (2.682%)	
**African American**	25 (12.255%)	1089 (6.838%)	107 (6.239%)	
**Unknown race**	26 (12.745%)	2973 (18.669%)	400 (23.324%)	
**Hypertension, N (%)**				< 0.001
**No**	109 (53.431%)	10,009 (62.851%)	815 (47.522%)	
**Yes**	95 (46.569%)	5916 (37.149%)	900 (52.478%)	
**Diabetes, N (%)**				< 0.001
**No**	171 (83.824%)	14,165 (88.948%)	1382 (80.583%)	
**Yes (Noninsulin)**	21 (10.294%)	1129 (7.089%)	193 (11.254%)	
**Yes (Insulin)**	12 (5.882%)	631 (3.962%)	140 (8.163%)	
**Smoking status, N (%)**				< 0.001
**No**	167 (81.863%)	12,893 (80.961%)	1316 (76.735%)	
**Yes**	37 (18.137%)	3032 (19.039%)	399 (23.265%)	
**Severe COPD, N (%)**				< 0.001
**No**	193 (94.608%)	15,266 (95.862%)	1569 (91.487%)	
**Yes**	11 (5.392%)	659 (4.138%)	146 (8.513%)	
**Congestive heart failure, N (%)**				< 0.001
**No**	201 (98.529%)	15,885 (99.749%)	1700 (99.125%)	
**Yes**	3 (1.471%)	40 (0.251%)	15 (0.875%)	
**Renal failure, N (%)**				0.086
**No**	203 (99.510%)	15,914 (99.931%)	1713 (99.883%)	
**Yes**	1 (0.490%)	11 (0.069%)	2 (0.117%)	
**Dialysis, N (%)**				< 0.001
**No**	203 (99.510%)	15,887 (99.761%)	1697 (98.950%)	
**Yes**	1 (0.490%)	38 (0.239%)	18 (1.050%)	
**Steroid use for a chronic condition, N (%)**				< 0.001
**No**	175 (85.784%)	13,676 (85.878%)	1284 (74.869%)	
**Yes**	29 (14.216%)	2249 (14.122%)	431 (25.131%)	
**Disseminated cancer, N (%)**				< 0.001
**No**	174 (85.294%)	12,641 (79.378%)	1104 (64.373%)	
**Yes**	30 (14.706%)	3284 (20.622%)	611 (35.627%)	
**Preoperative systemic sepsis, N (%)**				< 0.001
**No**	187 (91.667%)	15,375 (96.546%)	1613 (94.052%)	
**SIRS**	16 (7.843%)	541 (3.397%)	101 (5.889%)	
**Septic Shock**	1 (0.490%)	9 (0.057%)	1 (0.058%)	
**30-day mortality events, N (%)**				< 0.001
**No**	193 (94.608%)	15,570 (97.771%)	1627 (94.869%)	
**Yes**	11 (5.392%)	355 (2.229%)	88 (5.131%)	

### The results of the univariate analyses using a binary logistic regression model

The univariate analysis was performed and indicated that the following significant variables in patients were positively associated with postoperative 30-day mortality: female (OR = 0.652, 95% CI: 0.540 to 0.788), 41–60 years old (OR = 2.730, 95% CI: 1.706 to 4.370), 61–80 years old (OR = 4.933, 95% CI: (3.119 to 7.803), > 81 years old (OR = 14.629, 95% CI: 8.708 to 24.576), had diabetes (Noninsulin-dependent) (OR = 1.538, 95% CI: 1.129 to 2.096), diabetes (Insulin-dependent) (OR = 2.587, 95% CI: 1.881 to 3.557), severe COPD (OR = 2.496, 95% CI: 1.830 to 3.405), CHF (OR = 7.157, 95% CI: 3.494 to 14.661), hypertension (OR = 2.246, 95% CI: 1.859 to 2.713), renal failure (OR = 10.509, 95% CI: 2.922 to 37.800), dialysis (OR = 6.348, 95% CI: 2.989 to 13.483), disseminated cancer (OR = 2.835, 95% CI: 2.347 to 3.425), steroid use (OR = 2.278, 95% CI: 1.850 to 2.806), preoperative SIRS (OR = 2.557, 95% CI: 1.824 to 3.585), preoperative septic shock (OR = 9.019, 95% CI: 1.943 to 41.874), hyponatremia group (OR = 2.372, 95% CI: 1.868 to 3.012), hypernatremia group (OR = 2.500, 95% CI: 1.349 to 4.631), BUN (OR = 1.043, 95% CI: 1.036 to 1.050), WBC (OR = 1.072, 95% CI: 1.055 to 1.089), PLT (OR = 0.998, 95% CI: 0.997 to 0.999), HCT (OR = 0.924, 95% CI: 0.909 to 0.940), BUN (OR = 1.043, 95% CI: 1.035 to 1.050), and Cr (OR = 1.241, 95% CI: 1.114 to 1.383). By comparison, participants who were Asian (OR = 0.757, 95% CI: 0.401 to 1.429), African American (OR = 0.878, 95% CI: 0.590 to 1.306), of unknown race (OR = 1.152, 95% CI: 0.916 to 1.450) and those who smoked (OR = 1.130, 95% CI: 0.900 to 1.419) or had an abnormal BMI (OR = 0.987, 95% CI: 0.972 to 1.002) were not associated with postoperative 30-day mortality (Table 2).

**Table 2:** The results of the univariate analysis

	Statistics	OR	95% CI	P value
Sex, N (%)				
**Male**	8492 (47.590%)	Ref.		
**Female**	9352 (52.410%)	0.652	(0.540, 0.788)	<0.00001
**Race, N (%)**				
**White**	12,701 (71.178%)	Ref.		
**Asian**	523 (2.931%)	0.757	(0.401, 1.429)	0.38976
**African American**	1221 (6.843%)	0.878	(0.590, 1.306)	0.51996
**Unknown race**	3399 (19.048%)	1.152	(0.916, 1.450)	0.22611
**Age range, N (%)**				
**18–40**	2836 (15.893%)	Ref.		
**41–60**	7412 (41.538%)	2.730	(1.706, 4.370)	0.00003
**61–80**	7001 (39.234%)	4.933	(3.119, 7.803)	< 0.00001
**> 81**	595 (3.334%)	14.629	(8.708, 24.576)	< 0.00001
**Diabetes, N (%)**				
**No**	15,718 (88.086%)	Ref.		
**Yes (Noninsulin-dependent)**	1343 (7.526%)	1.538	(1.129, 2.096)	0.00632
**Yes (Insulin-dependent)**	783 (4.388%)	2.587	(1.881, 3.557)	< 0.00001
**Smoking status, N (%)**				
**No**	14,376 (80.565%)	Ref.		
**Yes**	3468 (19.435%)	1.130	(0.900, 1.419)	0.29262
**Severe COPD, N (%)**				
**No**	17,028 (95.427%)	Ref.		
**Yes**	816 (4.573%)	2.496	(1.830, 3.405)	< 0.00001
**CHF, N (%)**				
**No**	17,786 (99.675%)	Ref.		
**Yes**	58 (0.325%)	7.157	(3.494, 14.661)	< 0.00001
**Hypertension, N (%)**				
**No**	10,933 (61.270%)	Ref.		
**Yes**	6911 (38.730%)	2.246	(1.859, 2.713)	< 0.00001
**Renal failure, N (%)**				
**No**	17,830 (99.922%)	Ref.		
**Yes**	14 (0.078%)	10.509	(2.922, 37.800)	0.00032
**Dialysis**				
**No**	17,787 (99.681%)	Ref.		
**Yes**	57 (0.319%)	6.348	(2.989, 13.483)	< 0.00001
**Disseminated cancer, N (%)**				
**No**	13,919 (78.004%)	Ref.		
**Yes**	3925 (21.996%)	2.835	(2.347, 3.425)	< 0.00001
**Steroid use, N (%)**				
**No**	15,135 (84.818%)	Ref.		
**Yes**	2709 (15.182%)	2.278	(1.850, 2.806)	< 0.00001
**Preoperative systemic sepsis N (%)**				
**No**	17,175 (96.251%)	Ref.		
**SIRS**	658 (3.688%)	2.557	(1.824, 3.585)	< 0.00001
**Septic Shock**	11 (0.062%)	9.019	(1.943, 41.874)	0.00499
**Na**				
**Normal serum Na group (Na ≥ 135, < 146)**	15,925 (89.246%)	Ref.		
**Hyponatremia group (Na < 135)**	1715 (9.611%)	2.372	(1.868, 3.012)	< 0.00001
**Hypernatremia group (Na ≥ 146)**	204 (1.143%)	2.500	(1.349, 4.631)	0.00359
**WBC**	9.524 ± 4.460	1.072	(1.055, 1.089)	< 0.00001
**HCT**	40.291 ± 4.806	0.924	(0.909, 0.940)	< 0.00001
**PLT**	244.222 ± 76.614	0.998	(0.997, 0.999)	0.00144
**BUN**	17.348 ± 8.155	1.043	(1.035, 1.050)	< 0.00001
**Cr**	0.863 ± 0.450	1.241	(1.114, 1.383)	0.00009
**BMI**	28.728 ± 6.624	0.987	(0.972, 1.002)	0.08094

### The results of the multivariate analyses using the binary logistic regression model

To explore the link between preoperative serum Na level and postoperative 30-day mortality, we established three models by binary logistic regression models. We discovered a significant link between preoperative serum Na and postoperative 30-day mortality in the three models (Table 3). In the nonadjusted model, we found that a increase of 1 mmol/L in preoperative Na was related to an 8.9% decrease in postoperative 30-day mortality (OR = 0.911, 95% CI:0.888 to 0.936), which was statistically significant. In the minimally adjusted model, when we only adjusted for sex and age ranges, each elevation in the preoperative Na unit may result in a 7.3% decrease in the postoperative 30-day mortality (OR = 0.927, 95% CI:0.902 to 0.952). The findings on the link between preoperative serum Na level and postoperative 30-day mortality obtained from the model were statistically significant. In the fully adjusted model, a increase of 1 mmol/L of preoperative Na was accompanied by a 3.3% decrease in 30-day postoperative mortality (OR = 0.967, 95% CI:0.941 to 0.994). The distribution of CIs suggested that the relationship between the Na level and the postoperative 30-day mortality obtained by the model was reliable (Table 3).

**Table 3:** The results of the multivariate analysis

Exposure	Model 1 (OR, 95% CI,P)	Model 2 (OR, 95% CI,P)	Model 3 (OR, 95% CI,P)
**Na**	0.911 (0.888, 0.936) < 0.00001	0.927 (0.902, 0.952) < 0.00001	0.967 (0.941, 0.994) 0.01588
**Na groups**			
**Hypernatremia group (Na ≥ 146)**	Ref	Ref	Ref
**Normal serum Na group (Na ≥ 135, < 146)**	0.400 (0.216, 0.741) 0.00359	0.431 (0.231, 0.803) 0.00805	0.621 (0.315, 1.224) 0.16846
**Hyponatremia group (Na < 135)**	0.949 (0.498, 1.808) 0.87345	0.864 (0.451, 1.658) 0.66079	0.831 (0.409, 1.687) 0.60853

Model 1 (nonadjusted model): not adjusted for any covariate. Model 2 (minimally adjusted model): adjusted for sex and age range. Model 3 (fully adjusted model): adjusted for sex, age range, diabetes, serum WBC, HCT, PLT, BUN and Cr, severe COPD, CHF, hypertension, renal failure, dialysis, steroid use for chronic condition, disseminated cancer, and preoperative systemic sepsis. OR, odds ratio; CI: confidence, Ref: reference

### The nonlinearity addressed by the generalized additive model

**Fig. 2:**
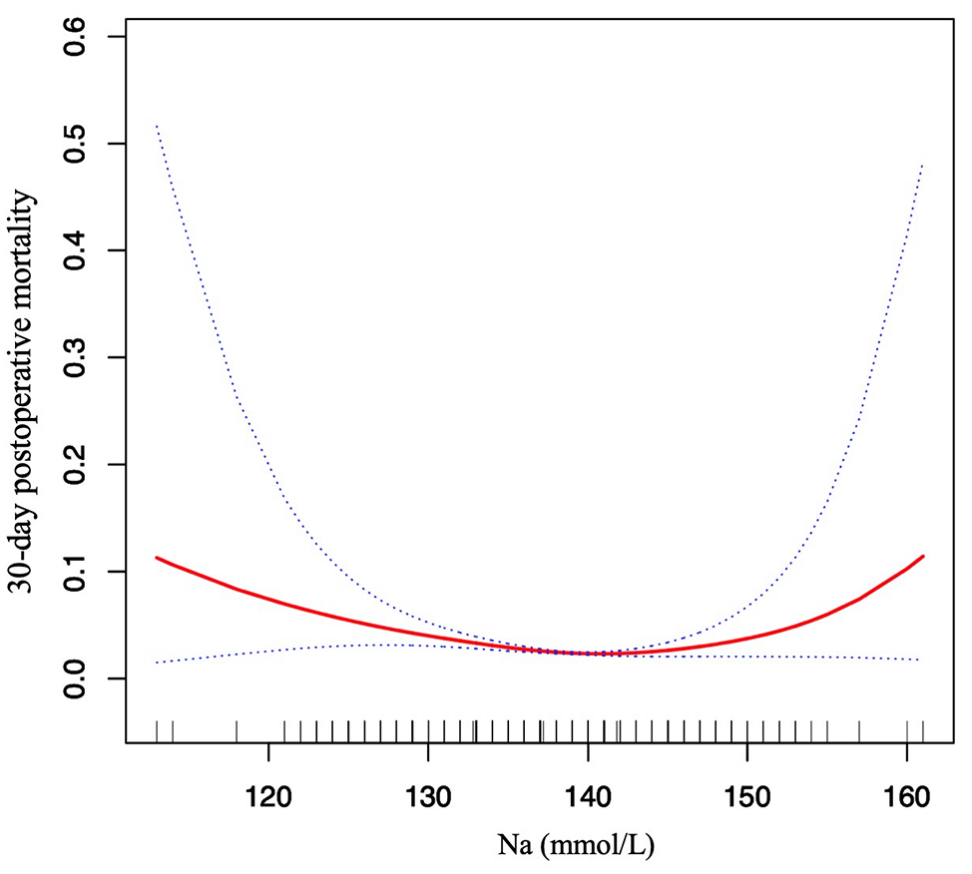
The nonlinear relationship between preoperative serum Na and 30-day postoperative mortality

We found that the relationship between preoperative serum Na and postoperative 30-day mortality was nonlinear through the GAM and smooth curve fitting (as shown in Fig. 2). Hence, we fit the data to a piecewise binary logistic regression model that allowed two different slopes and a standard binary logistic regression model based on the sensitivity analysis. The best fit model was chosen through the log-likelihood ratio test (as shown in Table 4). The P value for the log-likelihood ratio test was less than 0.05 in our analysis. Therefore, a piecewise model was used to fit the association between preoperative serum Na and postoperative 30-day mortality. With a recursive algorithm, we obtained an inflection point of 140 for the first time and computed the effect sizes (OR) and confidence intervals to the right and left of the inflection point with the piecewise binary logistic regression model. The effect size (OR) was 0.929, and the 95% CI was from 0.898 to 0.961 on the left side of the inflection point. The OR was 1.088, and the 95% CI was from 1.019 to 1.162 on the right side of the inflection point.

**Table 4:** The results of the standard linear and piecewise linear regression models

	Postoperative 30-day mortality (OR, 95% CI, P value)
**Fitting model by standard linear regression**	0.967 (0.941, 0.994) 0.0159
**Fitting model by two-piecewise linear regression**	
**Infection point of Na**	140
**≤ 140**	0.929 (0.898, 0.961) < 0.0001
**> 140**	1.088 (1.019, 1.162) 0.0118
**P value for the log likelihood ratio test**	< 0.001

The model adjusted for sex, age range, diabetes, serum WBC, HCT, PLT, BUN and Cr, severe COPD, CHF, hypertension, renal failure, dialysis, steroid use, disseminated cancer, and preoperative systemic sepsis. OR, odds ratio; CI: confidence, Ref: reference

### Subgroup analyses

We conducted subgroup analyses in view of other factors that might influence the link between preoperative serum Na level and postoperative 30-day mortality. We employed sex, severe COPD, hypertension, steroid use and disseminated cancer as stratification variables to test the trend of effect sizes. The results revealed that all listed subgroups did not affect the link between serum Na level and postoperative 30-day mortality (P for interaction > 0.05) (as shown in Table 5).

**Table 5:** Results of the interaction analysis and subgroup analysis

Characteristic	No of participants	OR (95% CI)	P for interaction
**Sex**			0.5130
**Male**	8492	0.960 (0.927, 0.994)	
**Female**	9352	0.977 (0.937, 1.020)	
**Severe COPD**			0.9466
**No**	17,028	0.965 (0.937, 0.994)	
**Yes**	816	0.962 (0.884, 1.047)	
**Hypertension**			0.4143
**No**	10,933	0.954 (0.913, 0.996)	
**Yes**	6911	0.976 (0.943, 1.011)	
**Disseminated cancer**			0.4315
**No**	13,919	0.974 (0.940, 1.010)	
**Yes**	3925	0.953 (0.915, 0.993)	
**Steroid use for a chronic condition**			0.7373
**No**	15,135	0.969 (0.938, 1.002)	
**Yes**	2709	0.960 (0.914, 1.007)	

### Sensitivity analyses

We conducted sensitivity analyses to test the robustness of our results. We first converted the preoperative Na value from a continuous variable to a categorical variable (dividing the patients into three groups based on hyponatremia and hypernatremia) and then changed the previous Na variable in the model to the categorical-transformed Na. The trend of the effect sizes in the different groups was equidistant after serum Na was converted to a categorical variable. Second, we performed a GAM to insert the continuity covariate into the equation as a curve. In addition, to assess the sensitivity to unmeasured confounders, we also calculated an E-value and obtained 1.21, which was greater than the relative risk of unmeasured confounders influencing the link between serum Na level and postoperative 30-day mortality, indicating that unmeasured or unknown confounders had little effect on the link between them.

## Discussion

Numerous studies have focused on the relationship between postoperative sodium disturbance and the outcome of brain tumor surgery. However, few studies have concentrated on the link between preoperative serum Na levels and the outcomes of brain tumors. Muhlestein WE et al. suggested that preoperative sodium abnormality was one of the strongest variables that influenced inpatient length of stay after brain tumor surgery [35]. In addition, Sorba EL et al. maintained that lower preoperative serum Na was an independent risk factor for SIADH after pituitary surgery [36]. These studies showed that preoperative abnormal blood Na levels may be a potential indicator for patient prognosis.

The authors used the ACS NSQIP database (2012–2015) to investigate whether there was a link between preoperative serum Na level and postoperative 30-day mortality in the present study. To the best of our knowledge, the present data analysis is the first to observe a nonlinear link between them in tumor craniotomy patients. The authors used a two-piecewise linear regression model to elucidate a nonlinear link between the serum Na level and 30-day postoperative mortality and first obtained the inflection point (140) of Na after adjusting for confounders. When the preoperative serum Na was below 140 mmol/L, a increase of 1 mmol/L of Na was related to a 7.1% decrease in postoperative 30-day mortality (OR = 0.929, 95% CI:0.898, 0.961). However, when preoperative serum Na was > 140 mmol/L, each increase in Na unit was related to a 8.8% increase in postoperative 30-day mortality (OR = 1.088, 95% CI:1.019, 1.162). Hyponatremia was associated with significant morbidity and mortality in a broad variety of malignant tumors [37], and hypernatremia at admission could be a meaningful indicator of poor outcomes in patients with terminal cancer [38]. These factors may explain the nonlinear association between serum Na level and postoperative 30-day mortality in our study. This is the first study to provide an inflection point as a reference for the management of preoperative serum Na in tumor craniotomy patients in the U.S. population. Hence, these results have prominent clinical value.

Our study has the following strengths. It is the large sample size that allows such analysis. Most covariates have complete information, with few missing. To the best of our knowledge, this is the first study to observe the association between preoperative serum Na levels and 30-day postoperative mortality in adult patients with tumor craniotomy. We found a nonlinear relationship between them, so our study has greater clinical significance, which previous studies have not investigated. We employed an interaction analysis and subgroup analysis. We used sensitivity analyses to test the robustness of our results.

Our research has the following shortcomings: this was a retrospective study on a national database; because our research was a secondary analysis of previously published data, we could not exclude some residual and/or unmeasured confounding factors that could interfere with the estimated link (e.g., pharmacological treatments, dietary habits, socioeconomic factors, pathological classification, perioperative fluid therapy, characteristics of benign and malignant tumors, and different types of brain tumors). The database used in this study lacked preoperative blood potassium and chlorine data. However, we computed the E-value to quantify the potential influence of unmeasured confounders, and we could not explore the link between preoperative serum Na level and long-term prognosis. Despite the above limitations, this research used collected data from a heterogeneous and large group of brain tumor patients. Therefore, the association and results postulated remain highly plausible. Besides, the database did not provide information about the clear cause of death. Future studies should collect those mentioned possible confounders and data that were not included in this study.

## Conclusions

This study proves a positive and nonlinear association between preoperative serum Na level and postoperative 30-day mortality in adult patients with tumor craniotomy and a threshold effect between them. These findings are expected to provide clinicians (especially neurosurgeons) with a suitable reference for preoperative serum Na. The use of the sodium inflection point of 140 mmol/L and proper preoperative management could reduce 30-day postoperative mortality after surgery in this population. Consequently, abnormal preoperative serum Na signals may assist clinicians in identifying high-risk groups for 30-day postoperative mortality in this particular population, which will facilitate clinicians planning and initiating appropriate management strategies.

## Data Availability

Anyone can download the raw data from https://journals.plos.org/plosone/article?id=10.1371/journal.pone.0235273.

## Abbreviations

Na: sodium
HCT: hematocrit
SD: standard deviation
CI: confidence interval
OR: odds ratio
ACS NSQIP: American College of Surgeons National Surgical Quality Improvement Program
BMI: body mass index
COPD: chronic obstructive pulmonary disease
GAM: generalized additive model
CHF: congestive heart failure
BUN: blood urea nitrogen
Cr: creatine
WBC: white blood cell count
PLT: platelet count
